# Tinea capitis in an immigrant pediatric community; a clinical signs-based treatment approach

**DOI:** 10.1186/s12887-021-02813-x

**Published:** 2021-08-26

**Authors:** Riad Kassem, Yahel Shemesh, Orna Nitzan, Maya Azrad, Avi Peretz

**Affiliations:** 1 Dermatology Department, Sheba Medical Center, Tel Aviv, Israel; 2 Sackler Faculty of Medicine, Tel Aviv University, Tel Aviv, Israel; 3 The Azrieli Faculty of Medicine, Bar-Ilan University, Safed, Israel; 4 Infectious Disease Unit, Baruch Padeh Medical Center, Poriya, Israel; 5 Clinical Microbiology Laboratory, Baruch Padeh Medical Center, Poriya, Israel

**Keywords:** Tinea capitis, Dermatophyte, Children of refugees, Empiric treatment

## Abstract

**Background:**

Tinea capitis is a common cutaneous infection of the scalp and hair follicles, typically diagnosed by direct examination and culture. Treatment with oral antifungals is usually withheld until mycology results are available. In Israel, African refugee children demonstrate higher susceptibility to Tinea capitis and generally fail to undergo follow-up evaluations.

**Methods:**

This study aimed to identify the clinical characteristics and treatment responses of refugee children in Israel with Tinea capitis, in order to formulate a treatment plan for primary care physicians. To this end, demographic, clinical and laboratory data were extracted from the electronic medical records of 76 refugee children presenting with Tinea capitis during 2016–2017. All measured variables and derived parameters are presented using descriptive statistics. The correlation between background clinical and demographic data and Tinea capitis diagnosis was assessed using the chi-squared and Wilcoxon tests. Correlations between demographic/clinical/laboratory characteristics and other types of fungi or other important findings were assessed using a T-test.

**Results:**

Scaling was the most common clinical finding. Cultures were positive in 64 (84%) and direct examination in 65 (85%) cases, with a positive correlation between the methods in 75% of cases. The most common fungal strain was *T. violaceum*. Fluconazole treatment failed in 27% of cases. Griseofulvin 50 mg/kg/day was administered to 74 (97%) children, and induced clinical responses. No side effects were reported.

**Conclusions:**

The key aim of this study was to emphasize the importance of diagnosis and treatment of these immigrant children by their primary pediatric doctor since it takes, an average of 4.3 months until they visit a dermatologist. During this critical time period, the scalp can become severely and permanently damaged, and the infection can become systemic or cause an outbreak within the entire community. In conclusion, we recommend to relate to scaly scalp in high-risk populations as Tinea capitis, and to treat with griseofulvin at a dosage of up to 50 mg/kg/day, starting from the first presentation to the pediatrician.

## What is Known

- Tinea capitis is a contagious infection, common among refugee children.
- Treatment with oral antifungals is usually withheld until mycology results are available.
- This is a critical period, because these children may infect others, and trigger epidemics in their densely populated communities.

## What is New

- In African refugee children with scalp scales suspected of Tinea capitis, a culture and direct examination and empiric treatment with griseofulvin 50 mg/kg/day are recommended at first presentation to the primary physician.

## Background

Tinea capitis is an infection of the scalp and hair follicles, caused by dermatophytes [1]. Dermatophytes is a common name of three genera of fungi – *Trichophyton*, *Microsporum* and *Epidermophyton*, with the first two species being most dominant. Dermatophytes are also classified by host preference and natural habitat, with anthropophilic dermatophytes affecting humans, zoophilic dermatophytes affecting animals, and geophilic dermatophytes mainly affecting the soil. The latter two are relatively rare [2, 3]. All dermatophytes can exploit keratin (hair, skin, and nails) for growth and their clinical manifestations are named after the affected area of the body (e.g., Tinea manum, Tinea pedis, Tinea capitis) [4]. Tinea capitis is one of the most common cutaneous infections in pre-pubertal children [1, 5–8], mainly from 6 months to 10–12 years of age [6]. The epidemiology of Tinea capitis varies across geographical regions throughout the world and changes over time [8, 9]. The infection spreads among family members and classmates^1^.

The clinical manifestations of Tinea capitis are variable, depending on the type of hair invasion, the level of host resistance, the immune system and the degree of inflammatory host response [6, 7], but can grossly be classified as alopecic or inflammatory. Inflammatory Tinea capitis includes kerion, which manifests as a tender mass with pustules, purulent discharge, lymphadenopathy, malaise, fever, and favus, with yellow cup-shaped crusts around the hair, inflammation and scarring [3, 10].

Tinea capitis is often misdiagnosed, thus delaying appropriate treatment and enabling spread of infection [7]. The diagnosis can be made in several ways. First and foremost, anamnesis and physical examination are crucial. In recent years, the dermatoscope has become more commonly used for close inspection of the scalp [11]. Diagnosis can be confirmed by direct microscopy, with use of standard potassium hydroxide (KOH) preparations. Its disadvantages include the time-consuming process, requirement of an expert and equipment not necessarily available in every clinic, and a false negative rate of up to 40% [12]. In addition, scalp scrapings and hair fragments from the affected area can be cultured, but can involve up to 3 weeks until results are provided [6, 12]. Molecular techniques such as polymerase chain reaction (PCR), provide for faster and more accurate identification of dermatophyte infections [6].

Systemic antifungal agents are indicated for all cases of Tinea capitis. While topical agents do not penetrate the hair shaft [7, 13], they are used as adjuncts to reduce the risk of transmission [1]. The common drugs in use are griseofulvin, terbinafine, itraconazole and fluconazole. The type and duration of treatment, as well as the dosage are determined by the dermatophyte strain [1, 2, 6, 14].

Refugee communities are generally of a low socioeconomic status and are associated with dense living conditions, which facilitate the spread of Tinea infections. In Israel, although refugees are medically insured up to the age of 18, they often receive suboptimal Tinea capitis care, partly due to the low availability of dermatologists in the southern part of Tel-Aviv, where most of them reside. Most cases are treated by pediatricians or family doctors, who generally have minimal expertise with this indication. As a result, the infection is frequently misdiagnosed, and when properly diagnosed, topical treatments are prescribed, which are rarely effective.

Treatment of Tinea capitis in refugee children poses a challenge, since optimal treatment demands repeated lab exams, yet compliance with such measures is very low because most parents work long hours and cannot afford to miss work days. In addition, a child diagnosed with Tinea capitis is forbidden to return to his daycare facility for several weeks, which requires that a parent miss more workdays. Thus, our aim was to simplify the diagnosis of Tinea capitis by primary physicians and speed initiation of appropriate treatment, by developing tools to accurately identify and treat the disease based on clinical finding and on physical examination. To this end, the clinical and demographic characteristics of 76 Tinea capitis-infected pediatric refugees living in the southern district of Tel Aviv, Israel, were analyzed and compared to the current knowledge in the field.

## Methods

### Study design

This was a retrospective study, conducted in the district of Tel-Aviv, Israel. The study analyzed the records of children aged 0–8 years (2 months – 7.7 years) with a clinical manifestation or positive culture of Tinea capitis, who were referred to a dermatologist in a secondary referral clinic of the Meuhedet sick fund and treated by the same dermatologist between January 2016 and December 2017. Data extracted from patient electronic records included: age, sex, country of birth, origin of parents, duration of lesion until contact with the dermatologist, infected family member (yes/no), high IgE level (yes/no), eosinophil blood count, history of immunosuppression, medical history, lesion description (alopecia, scaling, pruritus) and morphology, presence of kerion and lymphadenopathy, culture results, outcome of microscopic examination of skin scrapings and hair fragments (KOH exam), topical treatment (yes/no) and type and duration of systemic treatment.

### Statistical analysis

All measured variables and derived parameters are presented using descriptive statistics. The correlation between background clinical and demographic data and Tinea capitis diagnosis was assessed using the chi-squared and Wilcoxon tests. Correlations between demographic/clinical/laboratory characteristics and other types of fungi or other important findings were assessed using a T-test.

All tests were two-tailed, and a *p*-value of 5% or less was considered statistically significant. The data were analyzed using SAS® version 9.1 (SAS Institute, Cary, North Carolina).

## Results

The medical records of 76 children fulfilling the eligibility criteria were reviewed (Table 1). Out of 76 cases, 55 (72.4%) were boys and 21 (27.6%) were girls. The average age was 3.1 years (range 0–8 years). In total, 30 children (39.9%; 9 girls (30%) and 21 boys (70%)) had a family member infected with Tinea capitis. All children were born in Israel to parents who emigrated from Eritrea. The average time from the initial appearance of the lesion until contact with a dermatologist was 4.3 months (3 days - 24 month).

**Table 1:** Demographic and baseline characteristics of pediatric patients with suspected tinea capitis

Criteria	All cases n (%)	Culture		Direct examination	
		Positive n (%)	Negative^**a**^ n (%)	Positive n (%)	Negative n (%)
**Sex**	**76**	**64 (84)**	**12 (16)**	**65 (85)**	**11 (15)**
Male	55 (72.4)	46 (84)	9 (16)	49 (89)	6 (11)
Female	21 (27.6)	18 (86)	3 (14)	16 (76)	5 (24)
**Age (years)**	**3.3**				
0–2	13 (17.1)	10 (77)	3 (23)	12 (92)	1 (8)
2.1–4	38 (50)	31 (81)	7 (9)	33 (87)	5 (13)
4.1–6	24 (31.6)	22 (92)	2 (8)	19 (79)	5 (21)
6.1–8	1 (1.3)	1 (100)	0	1 (100)	0
**Eosinophilia**	23 (30)	21 (91)	2 (9)	21 (91)	2 (9)
**High IgE levels**	8 (10)	8 (100)	0	8 (100)	0

^a^ Contaminated culture was considered negative

**Fig. 1:**
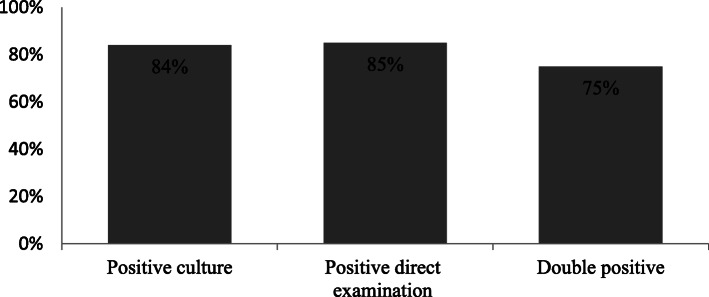
Percentage of positive tests for Tinea capitis out of all cases: 57 cases (75%) had both a positive culture and direct examination, while 72 cases had at least one positive test

Out of 76 cases, 64 had a positive culture, 18 of which were collected from girls (28%) and 46 from boys (72%). The remaining 12 cultures were contaminated. In parallel, 65 cases had a positive direct examination, 8 of which showed negative cultures (Table 1). Thus, 57 cases had both a positive culture and direct examination, while 72 patients have at least one positive test (Fig. 1).

The most common clinical manifestation among patients with a positive culture or a positive direct examination was scaling, and the least common was kerion with lymphadenopathy (Table 2). Kerion with lymphadenopathy provided the highest diagnostic specificity, while scaling was associated with the lowest diagnostic specificity. No single or combination of symptoms provided both high diagnostic sensitivity and specificity (Tables 2 and 3). Pruritus, as well as kerion with lymphadenopathy had a high diagnostic sensitivity but low specificity.

**Table 2:** Single symptoms at enrollment: relationship to positive dermatophyte culture (DC) and to positive direct examination (PDA) ^a^

Symptom	n/N		Sensitivity (%)		Specificity (%)		PPV (%)	NPV (%)		
	DC	PDA	DC	PDA	DC	PDA	DC	PDA	DC	PDA
Scaling	58/64	63/65	90.63	96.92	8.33	45.45	84.06	91.3	14.29	71.43
Alopecia	25/64	28/65	39.06	43.08	33.33	54.55	75.76	84	9.30	13
Pruritus	5/64	8/65	7.81	12.31	66.67	90.91	55.56	88.89	11.94	14.93
Kerion with lymphadenopathy	5/64	5/65	4.69	7.69	83.33	100	60	15.49	14.08	21.05

^a^ Contaminated culture was considered negative

**Table 3:** Multiple symptoms at enrollment: relationship to positive dermatophyte culture (DC) or to positive direct examination (PDA) ^a^

Symptom (pruritus, scaling, alopecia and kerion with lymphadenopathy)	n/N		Sensitivity (%)		Specificity (%)		PPV (%)	NPV (%)		
	DC	PDA	DC	PDA	DC	PDA	DC	PDA	DC	PDA
No symptoms	3/64	0/65	4.69	0	100	72.73	100	0	16.44	10.96
Any 1 symptom	34/64	31/65	53.13	47.69	88.33	54.55	94.44	86	25	15
Any 2 symptoms	24/64	29/65	37.50	44.62	41.67	81.82	77.42	20	11.11	50
Any 3 symptoms	3/64	5/65	4.69	7.69	75	90.91	50	83.33	15.79	14.29

**Fig. 2:**
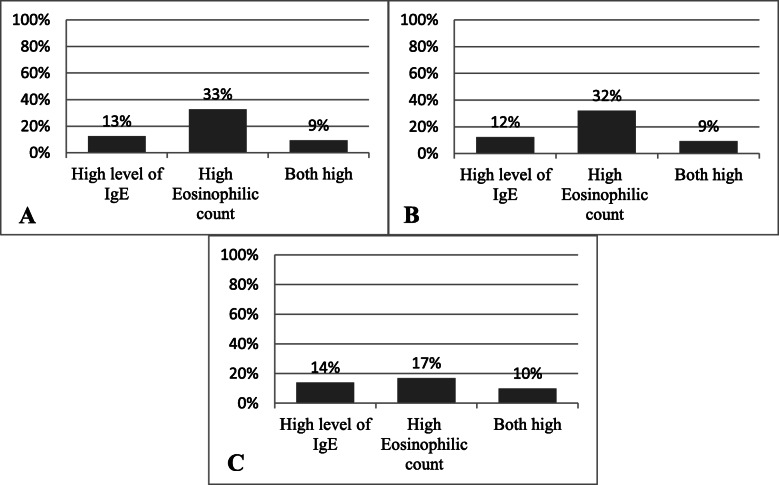
Percentage of children with high levels of IgE and/or eosinophilia out of all cases with positive A. Culture B. Direct examination C. Culture and direct examination

Out of the 64 children with a positive culture, 13 and 33% had high levels of IgE and eosinophilia, respectively (Fig. 2a) and 9% had both high IgE levels and a high eosinophilic count. Similar percentages of high IgE and/or eosinophilia were seen among the children with a positive direct examination (Fig. 2b). Among children with both a positive culture and a positive direct examination, fewer (17%) had eosinophilia as compared to those with only one positive test (Fig. 2c).

**Fig. 3:**
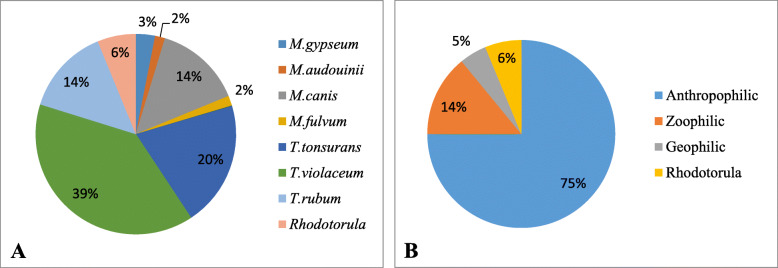
A. Percentage of the different types of dermatophyte fungi in culture among children with suspected tinea capitis. B. Percentage of the different types of dermatophyte fungi by host preference

The most common cultured dermatophyte species was *Trichophyton violaceum*, which was found in 25 (39%) samples. Out of the positive cultures, *T. violaceum* was found in 33% of samples collected from girls versus 41% of the samples collected from boys. The distribution of identified fungal species is shown in Fig. 3a. Distribution by host preference is presented in Fig. 3b.

Tinea capitis cases in children in this community prior to this study, were treated with griseofulvin as first-line therapy at a maximum dose of 25 mg/kg/day for 6–8 weeks, which induced a minimal response, if any. In order to eradicate and prevent spread of the infection, the recommended drug dose was gradually elevated to a dose of 50 mg/kg/day, for 6–8 weeks, which efficiently eradicated the infection. From then on, all children suspected to have Tinea capitis were treated with griseofulvin at this doubled dose.

During the study period, due to unavailability of griseofulvin for some time, 21 patients were treated with fluconazole, at a maximum dose of 7.5 mg/kg/day, for 4 weeks. None of these children showed a clinical response, even when the dose was raised to 10 mg/kg/day for 6 weeks. After the return of griseofulvin to the market, these children were treated with griseofulvin 50 mg/kg/day; clearance was achieved within 6 weeks in most cases, except for 3 children who required 8 weeks of treatment until the condition fully resolved.

Five children presented with kerion. In addition to antifungal treatment, they were treated with antibiotics and steroids. None required surgical intervention. Two children with both a negative culture and negative direct examination and suspected of having seborrheic dermatitis, were not treated empirically for Tinea capitis. Two other children with uncertainty regarding the possibility of Tinea capitis or seborrheic dermatitis diagnosis, were given empirical treatment, which was discontinued after receiving negative culture results.

Four children who showed no response to griseofulvin had a *R. mucilaginosa*-positive culture and were treated accordingly.

All children received topical treatment as adjunct therapy, as recommended, in order to reduce contagion. No clinical side effects or blood test abnormalities were observed in the participants during treatment. All treatments and responses are summarized in Table 4.

**Table 4:** Anti-mycotic treatment dosage and duration

Participants	Line	Drug	Dose	Duration (week)	Eradication (n/N)	Therapy replacement	Therapy stopped
53	1	Griseofulvin	50 mg/kg/day	8	49/53	4	1
18	2^a^			6	18/18	0	0
3				8	3/3	0	0
21	1	Fluconazole	7.5–10 mg/kg/day	10	0/21	20	1

^a^ Griseofulvin as second line was given after therapeutic failure with fluconazole

## Discussion

Since 2006, more than 60,000 people illegally emigrated from Africa to Israel through its border with Egypt, arriving with no belongings. Most of the refugees came from Eritrea, while others arrived from the Republic of Sudan and other African countries. This phenomenon increased considerably between 2007 and the end of 2012. A significant portion of this population settled in the southern Tel Aviv area. This population is generally of a low socioeconomic status, and resides in dense living conditions (data obtained from Tel Aviv municipality) [15]. The children are generally cared for at daycare centers, which can facilitate transmission, and are less likely to visit a tertiary clinic with expert dermatology services, and are therefore susceptible to Tinea capitis epidemics. Due to a focal outbreak of Tinea capitis among children in this community, and in order to prevent epidemics affecting other communities, this work attempted to characterize the clinical manifestations and provide primary physicians with appropriate diagnostic and management guidelines, to minimize the need for postponing the treatment until referral to a dermatology service.

In accordance with the literature regarding children of refugees of African descent in the US, Europe and Israel, *T. violaceum* was the most common cause of Tinea capitis [5, 8, 16]. Furthermore, 75% of positive cultures showed that the source of infection was an anthropophilic fungus, strengthening the conclusion that patients infect each other at daycare facilities and at home, due to the dense living conditions and lack of sufficient hygiene.

The probability of seeing an Israeli child with Tinea capitis is very low. Mashiah et al. who treated Tinea capitis in a pediatric population between the years 2011–2014 in a central tertiary reference center, found no cases through the year of 2010 [17]. Through the subsequent years, they started to gradually see more and more cases, which was ascribed to the increasing waves of refugees entering Israel. In this 4-year period, they had only 15 Israeli pediatric patients with Tinea capitis compared to 130 refugees [17]. The refugee community is estimated to include 60,000 individuals compared to 450,000 Tel-Aviv residents.

The most common clinical finding in children with either positive culture or positive direct examination was scaling, observed in 91 and 97% of cases, respectively, aligning with a previously published report [12]. However, in contrast to this earlier report, that dealt with urban hospital-based general pediatric practice, in the present study, the majority of children with a positive culture or positive direct exam had one positive sign, which was scales (53 and 48%, respectively). Moreover, the previous work found a high association between lymphadenopathy and positive Tinea capitis cultures, which stands in contrast with our results, where only 4.7% of the children with a positive culture and 7.7% of those with positive signs on direct examination, presented with lymphadenopathy. A different immune response to the same infection may underlie these conflicting observations, and may require an entirely different therapeutic approach.

In the current study population, many parents who visited a dermatologist with their infected child discontinued follow up at various stages, some after only one visit, likely due to long queues, distant clinics, loss of workdays and more. Some of the parents returned to the dermatologist only because the child was not allowed to return to the daycare center or due to development of infection and fever. Thus, treatment should be given to ensure suitable and efficient management from the initial and possibly only encounter with the patient.

In previously reported cases of Tinea capitis, insufficient response in skin of color populations was not solely due to lack of compliance, but rather, to a reduced clinical response of the fungi to the conventional griseofulvin doses [17]. As a result, over the past few decades, dosage elevations have been tested and have effectively achieved clearance. This might be due to suboptimal absorption of the drugs, different host response patterns to the same fungi or evolution of resistance to the drugs [17].

No specific association was observed between eosinophilia or IgE levels and susceptibility to Tinea capitis infection. However, this lack of correlation may have been the result of the small sample size. Alternatively, it may be due to subtle systemic signs in these children when compared to the other pediatric population, which may be rooted in differential immunologic responses to fungi or parasites, with the exception of kerion cases.

The four children who had no clinical response to griseofulvin, turned out to have a *R. mucilaginosa*-positive culture. Although rare, particularly in immunocompetent patients, scalp infection due to these unicellular pigmented yeasts that mimic Tinea capitis, has been observed in a refugee population [15]. Several therapeutic approaches have been described, including amphotericin B, ketoconazole, fluconazole, itraconazole and flucytosine, however, there is no consensus regarding the preferred treatment for such infections [15].

Taken together, for a refugee patient, a full physical examination should be performed. If findings like scaling, alopecia or pruritis are identified, the physician must rely on his highest degree of suspicion, and provide empirical treatment from the first encounter [18]. Prescriptions should be given for the entire treatment period, because it might be the only encounter with the patient.

Despite the challenges of follow up in this population, and in accordance with the literature, in any case of suspected Tinea capitis, the scalp should be cultured prior to treatment [2], at least for epidemiological investigation. If opportunity arises, follow-up with a repeat mycology culture at the end of treatment is recommended, as a definitive diagnosis of eradication [1].

Due to the relative resistance to traditional dosages of griseofulvin observed in this population and poor follow up, these patients should be treated at a higher dosage than usual. Although treatment decisions rely on the identity of the fungus [14, 18, 19], griseofulvin at a dosage of up to 50 mg/kg/day is recommended for first-line treatment, since it provides a sufficient clinical response. In addition, years of experience with the drug have demonstrated its long-term safety [13]; it has the fewest known drug interactions [17], a favorable adverse-effect profile [20] and rarely induces serious adverse-reactions [2]. Griseofulvin treatment has been associated with a small number of minor adverse effects, mainly gastrointestinal symptoms (vomiting, abdominal pain, diarrhea). Furthermore, it is the cheapest antifungal drug [13], a critical criterion for maintaining long-term compliance in the target population. When considering these benefits against the potential harm, empiric treatment prior to culture results is recommended [21]. As shown in Table 4, the study population seemed to be resistant to fluconazole. It is therefore not recommended to treat them with this drug, as it may result in lower compliance. Topical treatment alone failed to achieve clinical resolution, and therefore should only be administered as adjunct therapy. In case of an inflammatory lesion, such as kerion, additional treatment with steroids and antibiotic is needed. All the medications should be given together, to enhance compliance.

It is critical that the physician attempt to establish a relationship of trust with the parents in order to achieve whole-family care, to minimize reinfection cycles. It is also recommended to sterilize all shared hygiene instruments and wash bedding frequently. There might be a need for multidisciplinary collaborations (social worker, medical specialists) to coordinate and follow up on treatment plans and appointments. Mass testing in the neighborhood/schools may be warranted.

## Conclusions

The presented findings emphasized the importance of diagnosis and treatment of these immigrant children by their primary pediatric doctor since it takes, as mentioned, an average of 4.3 months until they visit a dermatologist. During this critical time period, the scalp can become severely and permanently damaged, and the infection can become systemic or cause an outbreak within the entire community. In conclusion, we recommend to relate to scaly scalp in high-risk populations as Tinea capitis, and to treat with griseofulvin at a dosage of up to 50 mg/kg/day, starting from the first presentation to the pediatrician.

## Data Availability

Not applicable.

## Abbreviations

IgE: Immunoglobulin E
*T. violaceum*: *Trichophyton violaceum*
